# 5-year trends in the intention to quit smoking amidst the economic crisis and after recently implemented tobacco control measures in Greece

**DOI:** 10.1186/1617-9625-12-S1-A17

**Published:** 2014-06-06

**Authors:** Sotiria Schoretsaniti, Filippos T Filippidis, Constantine I Vardavas, Christine Dimitrakaki, Panagiotis Behrakis, Gregory N Connolly, Yannis Tountas

**Affiliations:** 1 Center for Health Services Research, Department of Hygiene, Epidemiology and Medical Statistics,School of Medicine, National and Kapodistrian University of Athens, Athens, 11527, Greece; 2 Center for Global Tobacco Control, Department of Social and Behavioral Sciences, Harvard School of Public Health, West Boston, 02115, Massachusetts, USA; 3 Smoking and Lung Cancer Research Center, Hellenic Cancer Society, Athens, 11521, Greece; 4 Biomedical Research Foundation of the Academy of Athens, Athens, 115 27, Greece

## Background

The objective of the present study was to explore the trends in the intention to quit smoking among adults in Greece between 2006-2011, a period characterized by financial instability and newly endorsed tobacco control initiatives.

## Materials and methods

Trends analysis of 3 representative national and cross-sectional surveys, ‘Hellas Health I’ (2006), “Hellas Health III” (2010) and Hellas Health IV (2011).

## Results

Since 2006, the intention to quit smoking has significantly increased among both genders (33.3% [in 2006] to 42.4% [in 2011], p=0.002), among respondents aged >54 years (26.9% [in 2006] to 45.1% [in 2011], p=0.019) and among residents of rural areas (26.4% [in 2006] to 46.7% [in 2011], p=0.001). Both highest (32.1% [in 2006] to 49.4% [in 2011], p=0.036) and lowest (31.7% to 46.0%, p=0.021) socioeconomic (SE) strata showed an increase in the proportion of smokers who intend to quit. However, in 2011, quit attempts were more frequent (35.3%, p=0.009) in smokers of high socioeconomic status. Moreover, smoking prevalence has significantly decreased (43.1% [in 2006] to 38.1% [in 2011], p=0.023), mainly among men (52.4% to 45.7%, p=0.037), respondents of low socioeconomic status (38.9% to 29.4%, p=0.008) and residents of urban areas (45.2% to 37.9%, p=0.005).

## Conclusions

Over the past 5 years and possibly as a combined result of the implemented tobacco control policies and austerity measures, the intention to quit smoking has increased among all SE strata, however actual quit attempts were higher among those less disadvantaged. Further effort should be made to support quit attempts, especially among vulnerable populations.

